# A novel inhibitor of *Plasmodium falciparum* spermidine synthase: a twist in the tail

**DOI:** 10.1186/s12936-015-0572-z

**Published:** 2015-02-05

**Authors:** Pieter B Burger, Marni Williams, Janina Sprenger, Shaun B Reeksting, Mariëtte Botha, Ingrid B Müller, Fourie Joubert, Lyn-Marie Birkholtz, Abraham I Louw

**Affiliations:** Department of Biochemistry, UP Centre for Sustainable Malaria Control, Faculty of Natural and Agricultural Sciences, University of Pretoria, Private Bag X20, Hatfield, 0028 South Africa; Centre for Molecular Protein Science/Department of Experimental Medical Science, Lund University, S-221 00/84 Lund, Sweden; Department of Biochemistry, Bernard-Nocht Institute for Tropical Medicine, Bernard Nocht Strasse 74, Hamburg, D-20359 Germany; Swiss Federal Institute of Intellectual Property, Stauffacherstr. 65/59 g, CH-3003 Bern, Switzerland

**Keywords:** Spermidine synthase, *Plasmodium falciparum*, Virtual screening, Spermidine, Spermine, Pharmacophores, Crystal structures

## Abstract

**Background:**

*Plasmodium falciparum* is the most pathogenic of the human malaria parasite species and a major cause of death in Africa. It’s resistance to most of the current drugs accentuates the pressing need for new chemotherapies. Polyamine metabolism of the parasite is distinct from the human pathway making it an attractive target for chemotherapeutic development. *Plasmodium falciparum* spermidine synthase (*Pf*SpdS) catalyzes the synthesis of spermidine and spermine. It is a major polyamine flux-determining enzyme and spermidine is a prerequisite for the post-translational activation of *P. falciparum* eukaryotic translation initiation factor 5A (elF5A). The most potent inhibitors of eukaryotic SpdS’s are not specific for *Pf*SpdS.

**Methods:**

‘Dynamic’ receptor-based pharmacophore models were generated from published crystal structures of SpdS with different ligands. This approach takes into account the inherent flexibility of the active site, which reduces the entropic penalties associated with ligand binding. Four dynamic pharmacophore models were developed and two inhibitors, (1*R*,4*R*)-(*N*1-(3-aminopropyl)-trans-cyclohexane-1,4-diamine (compound 8) and an analogue, *N*-(3-aminopropyl)-cyclohexylamine (compound 9), were identified.

**Results:**

A crystal structure containing compound 8 was solved and confirmed the *in silico* prediction that its aminopropyl chain traverses the catalytic centre in the presence of the byproduct of catalysis, 5′-methylthioadenosine. The IC_50_ value of compound 9 is in the same range as that of the most potent inhibitors of *Pf*SpdS, *S*-adenosyl-1,8-diamino-3-thio-octane (AdoDATO) and 4MCHA and 100-fold lower than that of compound 8. Compound 9 was originally identified as a mammalian spermine synthase inhibitor and does not inhibit mammalian SpdS. This implied that these two compounds bind in an orientation where their aminopropyl chains face the putrescine binding site in the presence of the substrate, decarboxylated *S*-adenosylmethionine. The higher binding affinity and lower receptor strain energy of compound 9 compared to compound 8 in the reversed orientation explained their different IC_50_ values.

**Conclusion:**

The specific inhibition of *Pf*SpdS by compound 9 is enabled by its binding in the additional cavity normally occupied by spermidine when spermine is synthesized. This is the first time that a spermine synthase inhibitor is shown to inhibit *Pf*SpdS, which provides new avenues to explore for the development of novel inhibitors of *Pf*SpdS.

**Electronic supplementary material:**

The online version of this article (doi:10.1186/s12936-015-0572-z) contains supplementary material, which is available to authorized users.

## Background

*Plasmodium falciparum* is the most pathogenic of the human malaria species with approximately 207 million cases in 2012 and an estimated 627,000 deaths. The majority of the mortalities occur in Africa, mostly in children under the age of five and pregnant women. Anti-malarial drug resistance is a major concern especially against the artemisinins (the last remaining fully-effective anti-malarial) where resistance has recently been detected in Southeast Asia [1]. No new classes of anti-malarials have been introduced into clinical practice since 1996 and there is no vaccine available. A pressing need therefore exists to identify novel targets for new anti-malarial development [2].

The inhibition of polyamine biosynthesis has been widely studied as a target for antiproliferative therapy with some success in cancer prevention and treatment, but most notably in the treatment of West African sleeping sickness [3]. Polyamines are ubiquitous aliphatic amines that are essential for cell growth, proliferation and differentiation in the majority of living cells [4,5]. The major polyamines putrescine, spermidine and spermine are synthesized by ornithine decarboxylase (ODC, EC 4.1.1.17), spermidine synthase (SpdS; EC 2.5.1.16) and spermine synthase (SpmS, EC 2.5.1.22), respectively. The synthesis of spermidine and spermine requires decarboxylated *S*-adenosylmethionine (dcAdoMet) as aminopropyl donor, which is produced by *S*-adenosylmethionine decarboxylase (AdoMetDC, EC 4.1.1.50). The *P. falciparum* polyamine biosynthesis pathway has several unique and exploitable parasite-specific characteristics such as the association of the pathway-regulating enzymes, AdoMetDC and ODC, in a heterotetrameric bifunctional protein [6,7] and the absence of a polyamine interconversion pathway [7,8].

Accumulating evidence has highlighted the potential of several enzymatic activities involved in the *P. falciparum* polyamine pathway as targets for the development of anti-malarial chemotherapeutics [9,10]. The ensemble of polyamines increases during the asexual, intra-erythrocytic developmental cycle and occurs in millimolar concentrations within the parasite [11-13]. Spermidine levels of the intra-erythrocytic parasite exceed that of the other polyamines, emphasizing the role of *Pf*SpdS as a major polyamine flux-determining enzyme [11]. In addition, spermidine appears to have greater metabolic importance since it is a prerequisite for the post-translational activation of *P. falciparum* eukaryotic translation initiation factor 5A (elF5A), which is required for protein synthesis [9,14-17]. The biosynthesis of low concentrations of spermine has been attributed to a minor, secondary activity of *Pf*SpdS since there is no evidence for a *P. falciparum* equivalent to SpmS [18].

The crystal structures of several SpdS have been solved and released in the PDB, which include human, *Escherichia coli*, and plant SpdS [19]. The *Pf*SpdS structure was first described by Dufe *et al.* [20] and consists of two domains including an N-terminal β-strand (six antiparallel strands) and a central catalytic domain with a seven-stranded β-sheet flanked by nine α-helices forming a Rossmann-like fold, which is typical of methyltransferases and nucleotide-binding proteins. The active site is located between the N- and C-terminal domains and is divided into distinct binding cavities for its substrates dcAdoMet and putrescine, which is common for all SpdS. The active site is spanned by a so-called gate-keeper loop that is only structured when ligands are bound.

Several SpdS inhibitor studies have been performed in the last decades, with the most potent inhibitors of eukaryotic SpdS’s being two multi-substrate or transition state analogues, *S*-adenosyl-1,8-diamino-3-thio-octane (AdoDATO) and [3-(*R,S*)-(5′-deoxy-5′-carbaadenos-6′yl)-spermidine] (adenosylspermidine) [21], which bind to both substrate binding cavities. A potent inhibitor of *Pf*SpdS, *trans*-4-methylcyclohexylamine (4MCHA), was derived from a structure-activity relationship (SAR) study of the putrescine binding cavity, which highlighted the hydrophobic and hydrogen bond-donating pharmacophore features corresponding to the primary alkyl component and non-attacking nitrogen of putrescine, respectively [22]. The X-ray structure of the complex [PDB:2PT9] demonstrated that 4MCHA, only binds in the putrescine binding cavity when dcAdoMet is present [20]. In addition, 12 other crystal structures of *Pf*SpdS have been resolved including one co-crystallized with AdoDATO [PDB:2I7C] [20].

In the first structure-based drug design study of *Pf*SpdS, the information obtained from the crystal structure with AdoDATO was used to generate pharmacophore models [23]. Virtual screening of an in-house chemical library resulted in the identification of 28 compounds as active site binders but no significant inhibitors. In the present structure-based study a ‘dynamic’ receptor-based pharmacophore model was developed to identify potential inhibitors of *Pf*SpdS. This approach takes into account the inherent flexibility of the active site, which reduces the entropic penalties associated with ligand binding [24,25]. Subsequent co-crystallization of *Pf*SpdS with MTA and two potential inhibitors yielded one crystal structure with compound 8 that validated the *in silico* predicted interactions, i.e., the aminopropyl tails of these compounds cross the catalytic centre and bind into the aminopropyl cavity of the dcAdoMet site. However, the 100-fold better inhibition by compound 9 compared to compound 8 could only be explained by their binding in a reversed orientation in the presence of dcAdoMet with their aminopropyl tails facing the non-attacking side of the putrescine/spermidine binding cavity. Compound 9 is thus predicted to inhibit *Pf*SpdS by capitalizing on its ability to produce spermine, which concur with previous literature reports that compound 9 is an inhibitor of mammalian spermine synthase but not mammalian SpdS [22]. Therefore compound 9 acts as a spermidine analogue and specifically inhibits *Pf*SpdS due to its unique ability to also accept spermidine as substrate for spermine synthesis.

## Methods

### Protein structure quality assessment

The structural integrity and quality of the [PDB:2I7C] and [PDB:2PT9] *Pf*SpdS crystal structures were analysed with WHATIF [26] and PROCHECK [27]. pKa predictions were performed using UHBD [28] and YASARA [29] on the [PDB:2HTE] and [PDB:2I7C] *Pf*SpdS crystal structures and included the solvent molecules. The pKa predictions were performed by adjusting the default scripts in the UHBD package as follows: the pKa values were predicted at a pH of 7.0, the temperature was set to 293 K and the dielectric constants of the solvent and protein were kept at 80 and 20, respectively. Lastly, the protonation states of the His residues were assigned based on consensus decisions (His103HSD, His108HSE, His236HSE and His304HSE).

### Phase space sampling

The molecular dynamics (MD) simulations and energy minimization were performed with NAMD [30] using the CHARMM force field [31] in the absence of AdoDATO. The dimension of the water box was set to 79 × 100 × 81 Å with the water boundary set at a distance of 10 Å from the protein. In preparation for the *Pf*SpdS equilibration run the protein was protonated according to the pKa values obtained above. Steepest descent (SD) minimization consisting of 100 steps was performed on the added hydrogens followed by solvation and neutralization of the protein using VMD [32]. The water and ions in the water box were subsequently minimized and equilibrated. This was done for 2,000 steps of SD minimization followed by a 20 picosecond (ps) equilibration of the water and ions with a reassignment of velocities every 1 ps. This step was followed by minimization of the protein for 200 steps using SD while keeping the solvent fixed. The protein and solvent were then heated to 310 K with a heating gradient of 10 K every 500 steps (1 ps) after which the protein was kept at 310 K for 34 ps resulting in a total duration of the heating process of 50 ps. The protein was then equilibrated for 500 ps and the temperature reassigned every 500 steps to 310 K. Periodic boundary conditions (PBC) were applied and all the electrostatic interactions were included using the Particle Mesh Ewald (PME) summation method [33]. Constant pressure and temperature control was applied using the Berendsen method [34]. The production run was performed under PBC conditions. As with the equilibration run, Berendsen dynamics were used to perform constant pressure and temperature control.

The resulting trajectory was clustered with the gromos algorithm [35] of the g_cluster module from GROMACS [36] to obtain the central structure of the representative clusters. The clustering was performed separately for each monomer of the simulated dimer. The MD trajectory was aligned and clustered based on the active site of each of the monomers. The cut-off values used for the clustering were 1.15 Å and 1.14 Å for chain B and C, respectively. The isomiddle structures of the top five representative clusters of both monomers were selected and compared relative to root mean square deviation (RMSD). From these structures five were selected to best represent the RMSD range between the structures and subsequently used in further studies.

### Negative image construction and hit analysis

The sub-ensemble of structures selected in the previous section was prepared for molecular interaction field (MIF) analysis, which was performed with GRID [37]. A grid box was generated to cover the active site with the following dimensions: TOPX 19.82, BOTX 16.73; TOPY 16.64, BOTY 20.85; TOPZ 19.28 and BOTZ 14.02. Two water-binding hotspots were identified and subsequently added to the sub-ensemble of structures. MIF analysis was performed to explore the chemical nature of the active site by using probes representing hydrogen bond donors (HBD), hydrogen bond acceptors (HBA) and hydrophobic (HYD) PhFs. Three different HBD probes were used and included the N2 (a neutral flat nitrogen with two hydrogens (NH2)), N1+ (a sp amine (NH) cation) and N3+ (a sp amine (NH) cation) probes. For the identification of HBA binding hotspots three probes were used and included the O (a sp carbonyl oxygen), O1 (an alkyl hydroxy (OH) group) and N:= (a sp2 nitrogen with lone pair of electrons) probes. The HYD features were identified using both the DRY (a general hydrophobic probe) and Me (the C3 probe represents a CH_3_ chemical moiety) probes. The default GRID parameters were used except that the number of planes of grid points per Angstrom (NPLA) and ALMD parameters were changed to four (spacing the grid points 0.25 Å apart) and one (print additional data in the output files), respectively.

### Pharmacophore model selection, screening and docking

The binding energy values and coordinates for all the respective probes were extracted from the GRID calculations for each of the structures from the sub-ensemble and were represented in Cartesian space. Grid point clusters were identified and selected by visualization of the grid points within the active site. The PhFs were generated by extracting the attributes from the grid point clusters and calculating their centre of mass (energy-weighted) as well as their radius of gyration. This was performed on all the probes used in MIF analysis, using an in-house python script that generates a PDB file containing the centre of mass (representing the PhF coordinates; energy-weighted), the radius of gyration and the mean energy of the PhF. The PhFs identified for the sub-ensemble and those from the [PDB:2PT9] crystal structure were treated separately. Atoms to represent the exclusion volumes (EVs) for the DPM were selected after visual inspection of the residues surrounding the active site. The coordinates for these atoms were extracted from all the structures in the sub-ensemble. The centre of mass for these atoms was calculated to include the dynamic behaviour of the active site and thus represent ‘dynamic’ EVs, which was also performed using an in-house python script.

For the selection of DPMs the active site was subdivided into four regions, DPM1 to DPM4. For each of these regions various DPMs represented by different combinations of PhFs were constructed. The DPMs were built in Catalyst v4.10 (Accelrys). The EVs were added using Discovery Studio 2.0 before screening subsets of the ZINC database. The drug-like subset of the ZINC database was used to construct a multi-conformer composite database and was generated using catDB (Accelrys). During database construction the maximum number of conformers was limited to 250. Catalyst v4.10 was used to search both the constructed databases selecting the best flexible search parameter. The DPMs used during searching included the EVs generated from the sub-ensemble of *Pf*SpdS. Compounds identified during these searches were fitted to its DPM to get the best fitting molecules and these were ranked accordingly. Visual inspection of the best-fitting compounds was performed to select the top compounds based on their fit values and orientation within the active site. Selected compounds were initially docked using AutoDock v4.0. Two different *Pf*SpdS monomers were prepared for docking and included chain C of [PDB:2I7C] and chain A of [PDB:2PT9], following 400 steps of steepest decent energy minimization. The AutoDockTools (ADT) kit was used in preparation of both the target structures and identified compounds. The following parameters were changed for docking: the Genetic Search Algorithm was selected and the number of genetic algorithm runs changed to 50; the Translation parameter was changed to 0.2 (Å/step); both the Quaternion and Torsion parameters were changed to 5 (degree/step); and, the RMS Cluster tolerance to 1.5 Å. The docked compounds were evaluated based on their energy scores and poses within the active site of both the [PDB:2PT9] and [PDB:2I7C] *Pf*SpdS crystal structures used during docking.

**Figure 1:**
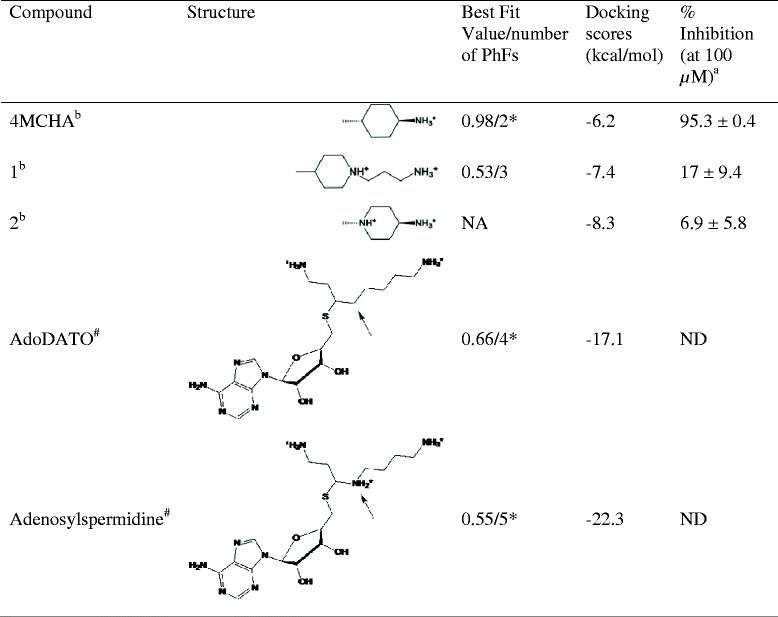
**Docking results of compounds identified from virtual screening and** ***in vitro*** **inhibition of** ***Pf*** **SpdS.** ^a^Results represent the percentage PfSpdS activity inhibited compared to untreated enzyme of three independent experiments performed in duplicate. Data are given as means ± SEMs. ^b^These compounds were docked in the presence of both MTA and dcAdoMet. NA; not applicable. ND; not determined. *Used as control to verify DPMs. ^#^Atoms predicted to influence binding affinity.

The binding poses of the selected compounds were refined using Glide (Schrödinger, Inc) as docking engine. The compounds were prepared for docking by predicting their protonation states at pH 7.4 by using the program Epik (Schrödinger, Inc.). The docking grids were generated for *Pf*SpdS ([PDB:2I7C]; chain B) using Asp196 as the centre with the grid size set to 33 Å, and the ligand diameter midpoint box was set to 14 Å for all three axes. The extra precision scoring algorithm from Glide was used to identify the best scoring compounds during docking. All ligands were treated as flexible during docking, allowing for sampling of nitrogen inversion and ring conformations. Docking poses were restricted to ten poses per ligand followed by postdocking minimization (minimization was performed with the Optimized Potentials for Liquid Simulations-all atoms force field) with a rejection threshold of 0.5 kcal/mol. The structures of the best-scoring docked pose for each of the compounds are denoted in Figure 1 and in Additional file 1. MM/GBSA calculations were performed using Prime to determine the binding free energies of selected ligands (Schrödinger, Inc). Protein flexibility was incorporated for residues within 12 Å of the selected ligands.

### Enzyme inhibition assays

Compounds tested for inhibition of *Pf*SpdS (Figure 1, Table 1 and Additional file 1) were supplied by the following manufacturers: compound 1 by Matrix Scientific (Columbia, South Carolina, USA), compound 2 (purity 97%) by CHEM IMPEX International, Inc (Wooddale, Illinois, USA), compounds 3, 4, 7 and 9 (purity 98%) by TCI Europe (Tokyo, Japan), compound 8 (purity 97.7%) was synthesized by PharmaAdvance Inc (China) and 4MCHA was a kind gift from Keijiro Samejima (Josia University, Saitama, Japan). A 29 amino acid N-terminus deletion mutant of *Pf*SpdS cloned into pTRCHisB (Invitrogen) was expressed in *E. coli* BLR (DE3), purified and assayed as described by Haider *et al*. [18]. In later studies, an improved developing medium for TLC separations consisting of iso-propanol, acetic acid and water saturated with NaCl (62.5:15:17.5, v/v/v), was employed. Initial inhibition assays were performed with 100 μM of each of the compounds before their selection for IC_50_ determinations. Stock solutions of the inhibitors were prepared in dddH_2_O. For the 100 μM assays, 1 μg purified *Pf*SpdS, 50 μM RS-dcAdoMet, 100 nC_i_ [2,3-^3^H] putrescine hydrochloride (60 C_i_/mmol; American Radiolabelled Chemicals, Inc, Minnesota, USA) and 50 μM unlabelled putrescine dihydrochloride (Sigma) were used. For IC_50_ determinations 0.5 μg purified *Pf*SpdS, 350 μM RS-dcAdoMet (5xK_m_) and 250 μM unlabeled putrescine hydrochloride (5xK_m_) and a range of concentrations of compound 9 (7.5-120 μM), compound 8 (125–1,000 μM), and 4MCHA (0.5-64 μM) were used for IC_50_ determinations. All inhibition assays were performed in triplicate.

**Table 1:** **Effects of various inhibitors on** ***Plasmodium falciparum*** **spermidine synthase**

	**Mammalian**	***P. falciparum***
**Inhibitor**	**Enzyme inhibition, IC**_**50**_ **(μM)**	**Enzyme inhibition, IC**_**50**_ **(μM)**
4MCHA	1.7^†^	1.4 ± 0.1^*^
Cyclohexylamine	8.1^§^	19.7 ± 3.1^*^
APE	1.7^┴^	6.5 ± 2.1^*^
AdoDATO	0.05-0.10^†^	8.5^#^
Adenosylspermidine	0.014^†^	N.D.
MTA	N.D	159 ± 27^*^
Compound 8	N.D.	619 ± 144^a^
Compound 9	N.D.	7.4 ± 2.4^a^

^a^Results are the mean of at least three independent, duplicate experiments ± SEM.

*Haider *et al.* [18].

^#^Dufe *et al.* [20].

^§^Shirahata *et al.* [22].

^†^Lakanen *et al. *[21]; Pegg *et al.* [38].

^┴^Goda *et al.* [39].

### Protein purification and crystallization of *Pf*SpdS

For protein crystallization of *Pf*SpdS the gene corresponding to a protein lacking 39 amino acids at the N-terminus, and cloned into the p15-TEV-LIC vector, was obtained from SGC Toronto [40]. As described by Dufe *et al.,* this truncation is crucial to obtain protein crystals that can be used for structure determination [20]. The protein expression and isolation was followed according to Dufe *et al.* [20]. His-tag cleavage of the purified protein with Pro-TEV protease (Promega) was performed overnight at 4°C in the presence of 1 mM DTT. The cleaved protein was purified with Ni-NTA (Sigma-Aldrich) affinity chromatography and buffer exchange was performed in crystal buffer (10 mM Hepes, pH 7.5, 500 mM NaCl). The protein was concentrated to 22.8 mg/mL and stored at 4°C.

*Pf*SpdS was crystallized in the presence of compound 8 and compound 9 as well as with and without MTA using the hanging drop vapour diffusion method at 293 K. Protein solution at a concentration of 5 mg/ml was mixed with reservoir solution containing 25% PEG3350, 0.1 M MES pH 5.6 and 0.1 M (NH_4_)_2_SO_4_. The *Pf*SpdS inhibitor-MTA crystals were obtained by pre-incubation of 5 mg/mL protein (1 *μ*L) with 2.5 mM of compound and MTA each for 30 min at 298 K followed by mixing with 1 *μ*L of reservoir solution. Crystals were transferred to cryo-protectant solution containing the reservoir solution and 15% glycerol prior to freezing directly in a nitrogen cryostream at 100 K. Data was collected at beamline I911-2 (MAX Lab, Lund, Sweden) and processed using the XDS package [41].

Molecular replacement was performed with CNS (v1.2) [42] using the *Pf*SpdS-MTA structure [PDB:2HTE] as search model with subsequent refinement using REFMAC [43] with model building in COOT [44]. The library files for compound 8 were generated using the PRODRG server [45]. The main chain polypeptide conformations of the crystal structures were verified by Ramachandran plots using RAMPAGE [46]. Model quality was checked with PROCHECK and the Joint Structural Genomics Consortium Quality Control v2.7, which contains MolProbity [47] and ADIT [48] checks.

## Results

### Protein phase space sampling and clustering

Phase space sampling was used to explore the protein conformational landscape in order to identify a diverse set of conformations over time representative of the active site in solution. An ensemble of protein binding sites has been shown to increase enrichment and identification of a diverse set of ligands when used in virtual screens over a single protein conformation [49]. In this study a MD simulation was used as the phase space sampling method to estimate the equilibrium and dynamic properties of the *Pf*SpdS dimer co-crystallized with AdoDATO [PDB:2I7C]. A 5 nanosecond (ns) MD simulation of the ligand bound enzyme showed similar RMSDs for subunits B and C in the first 2.5 ns (Additional file 2). There was a slight increase in the backbone RMSD of subunit C over the last 2.5 ns compared to subunit B due to increased movement of the gate-keeping loop. Subunits B and C were therefore treated as two separate trajectories and clustered accordingly. Clustering of the trajectory was based on 62 residues identified within 7 Å of AdoDATO in the binding cavity and used to obtain at least 90% of the MD trajectory frames into the five most populated clusters (93.4 and 99.1% for subunits B and C, respectively). The iso-middle structures of the top five clusters for both monomers were selected and their structural diversities were compared based on the RMSD values of their active sites (Table 2). Six of the ten structures were selected based on these RMSD values as representative of the sampled conformations (96.2% of the total sampled space) forming a sub-ensemble of structures (Table 2) and subsequently used for negative image construction.

**Table 2:** **Clustering of the sampled phase space**

**Protein subunit**	**C**_**α**_ **-backbone RMSD (Å)**	**Active site RMSD (Å)**	**% representation**
Cluster 1B^#,^*	0.00	0.00	67.74
Cluster 2B*	1.46	1.86	19.20
Cluster 3B*	1.19	1.32	6.62
Cluster 4B	1.45	1.89	0.24
Cluster 5B*	0.72	0.69	0.22
Cluster 1C*	1.67	2.25	34.24
Cluster 2C	1.56	1.99	26.32
Cluster 3C	1.59	2.00	19.32
Cluster 4C	1.38	1.74	6.69
Cluster 5C*	1.80	2.40	6.74
PDB:2PT9A	1.56	1.99	-
PDB:2PT9B	1.65	2.17	-
PDB:2PT9C	3.77	3.68	-

^#^Reference structure.

*Structures selected to represent the sub-ensemble.

The protein subunits represent the top five clusters of subunits B and C from MD. The representation of the sampled space of each cluster from the MD is given as a percentile of the total sampled space per subunit. The subunits of the ligand bound crystal structure [PDB:2PT9] with its C_α_-backbone and active site RMSD values are included.

Conformational changes within the putrescine-binding cavity were determined by comparing the starting structure [PDB:2I7C] and the sub-ensemble of structures. The most prominent feature noted is the conformational change of Gln229 in the absence of AdoDATO; the side-chain amide group orientates perpendicular in the ligand free state compared to the orientation with the ligand bound state, which corresponds to the crystal structure of the ligand free state [PDB:2PSS] (Additional file 3). A superimposition of the ligand free *Pf*SpdS sub-ensemble on the putrescine-bound human SpdS [PDB: 2O06] structure and the *Pf*SpdS crystal structures bound with AdoDATO as well as 4MCHA-dcAdoMet showed that Gln229 assumes a nearly identical orientation for all of these structures (Additional file 3). Importantly, this conformational change significantly alters the binding characteristics of the putrescine-binding cavity and introduces a series of caveats in the identification of pharmacophore features (PhFs) during negative image construction. Therefore, in addition to the sub-ensemble of structures, the three protein chains of *Pf*SpdS co-crystallized with 4MCHA and dcAdoMet [PDB:2PT9] were included in the negative image construction to identify PhFs representing both the ligand bound and ligand free states (Table 2). The 5 ns MD simulation is only able to sample a small part of the conformational energy landscape, however, in combination with the ligand-bound crystal structures it covers a diverse set of structural conformations [49].

### Negative image construction

The process of generating PhFs representative of small molecules using a protein structure is known as negative image construction. Probes descriptive of HBD, HBA or HYD characteristics are placed within the binding cavity and used to identify the most favourable binding hotspots for the PhFs. These PhFs are then selected to generate pharmacophore models that can be used in virtual screening of small molecule chemical libraries. Using this methodology a large number of PhFs were identified, which allowed the selection of a set of pharmacophore models to be used in virtual screening.

Energetically favourable binding hotspots were identified by molecular interaction field (MIF) analysis and used to derive the initial PhFs. These PhFs were compared to the known binding features obtained from the *Pf*SpdS structures co-crystallized with inhibitors. Eight different probes representing HBA, HBD and HYD characteristics were used in MIF analysis (see Methods). The resulting grid points representative of the favourable binding areas were extracted and overlaid. Clusters from these grid points were identified and selected to represent PhFs (binding hotspots) if they represented at least 60% of the sub-ensemble of structures. The binding energies associated with each of the grid points were assigned as a weight and the centre of masses were calculated to determine an energy-weighted centre of the cluster (ranging between −1.3 for the HYD probes to −24.0 kcal/mol for the HBD probes). This process improved the representation of the binding hotspot defined by the PhF. In addition, the radius of gyration was calculated and used as a guide to determine the radius of the PhFs (0.4 - 2.5 Å).

As a control the PhFs identified during MIF analysis were compared with known PhFs derived from published crystal structures with bound inhibitors and two PhFs were found to have moved ~2 Å within their respective binding pockets. These PhFs represent the aminopropyl nitrogen group of dcAdoMet and the nitrogen of 4MCHA (corresponding to the non-attacking nitrogen of putrescine). Inspection of two crystal structures [PDB:2I7C] and [PDB:2PT9] revealed the interaction of two solvent molecules with chemical moieties binding within these PhFs. The coordinate positions for these water molecules were determined using a water probe and subsequently added to the structures and energy optimized prior to MIF analysis.

EVs i.e. atoms within 7 Å of AdoDATO co-crystallized with *Pf*SpdS [PDB:2I7C], were specified to facilitate database searching. The atomic coordinates of the selected EVs within the sub-ensemble of structures were extracted and their centres of masses were calculated to incorporate target flexibility (in-house python script). These ‘dynamic’ EVs are improved representations of the boundaries of the active site since they indicate an average position of the atoms over time. The EVs were overlaid with the co-crystallized ligands AdoDATO, dcAdoMet and 4MCHA to identify steric clashes, which were subsequently removed.

### Pharmacophore model selection

**Figure 2:**
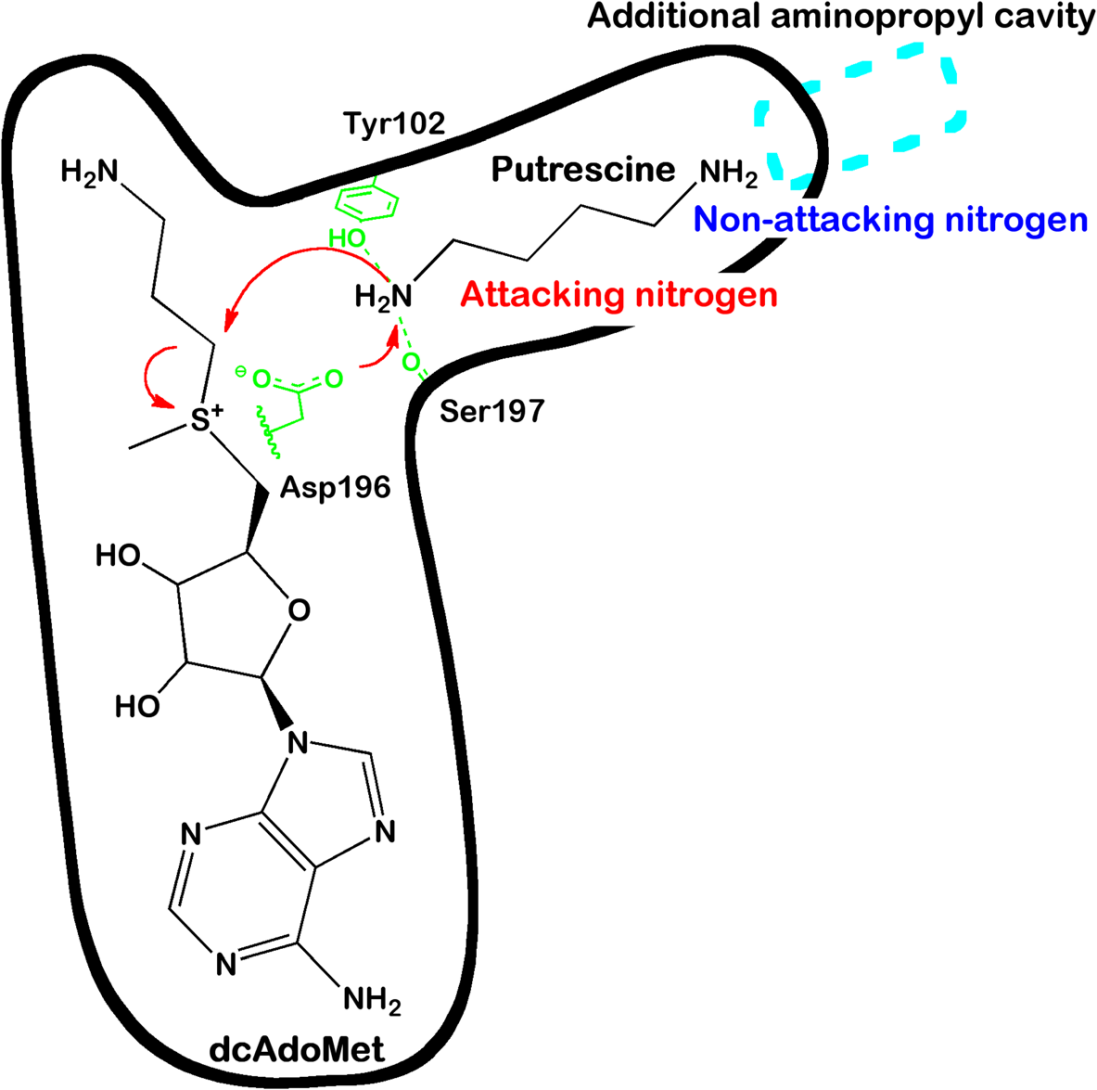
**A representation of putrescine and dcAdoMet within their binding cavities.** Residues important for catalysis are shown in green. Red arrows indicate the proposed attack by the putrescine amino group (attacking nitrogen) on the methylene carbon of the aminopropyl chain of dcAdoMet resulting in the catalysis that produces spermidine via a SN_2_ reaction. Highlighted in cyan is the aminopropyl cavity (in the vicinity of the non-attacking nitrogen of putrescine) that can be accommodated by the aminopropyl chain of spermidine to catalyze the production of spermine.

**Figure 3:**
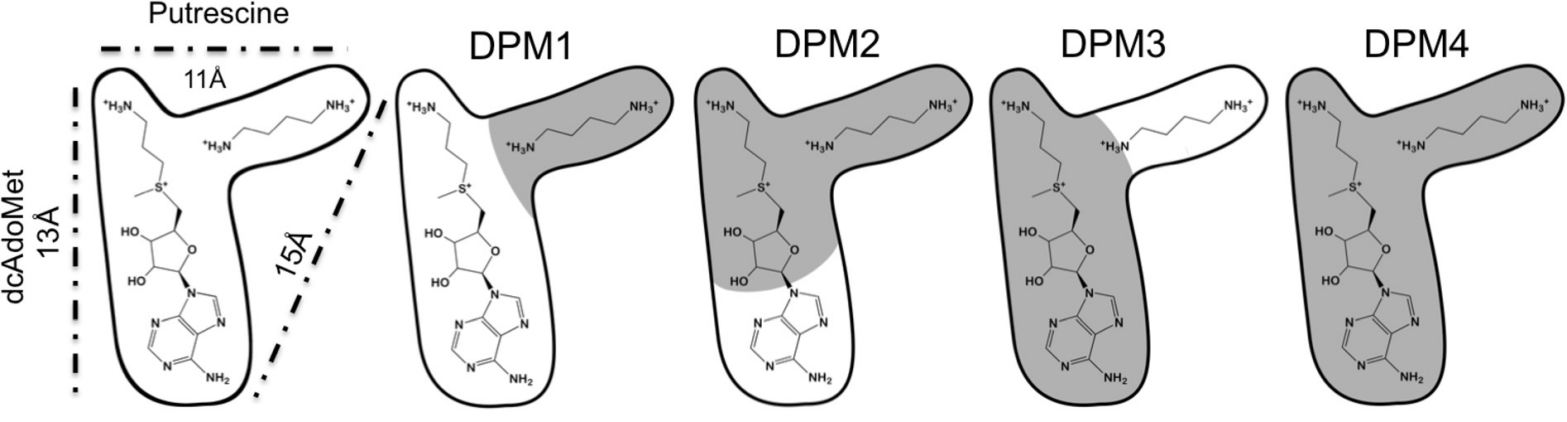
**A representation of the r-shaped** ***Pf*** **SpdS active site and its natural substrates, dcAdoMet and putrescine.** The distances (Å) between the PhFs furthest apart and representative of the dimensions of the active site are shown. The shaded region of the active site indicates the DPM binding regions used in the virtual screen.

The active site of SpdS contains binding cavities for dcAdoMet and putrescine [50,51]. The putrescine binding cavity of *Pf*SpdS also accommodates the aminopropyl chain of spermidine at its non-attacking end when spermine is synthesized [18,20] (Figure 2). Figure 3 shows the subdivision of the r-shaped active site into four binding regions with the native substrates within their respective binding cavities for dynamic pharmacophore models (DPM1 to DPM4) to facilitate pharmacophore searches and explore specific regions within the active site. The DPM1 binding region was selected to explore the putrescine-binding cavity; the DPM2 binding region was selected to explore compounds that protrude from the putrescine-binding cavity into the dcAdoMet cavity; the DPM3 binding region was selected to explore compounds that protrude from the dcAdoMet-binding cavity into the putrescine binding cavity; while the DPM4 binding region was used to explore the entire active site (Figure 3).

### DPM1 binding cavity

Two PhFs that best described the receptor-derived pharmacophore model for 4MCHA were used to verify the model’s ability to identify this inhibitor. The basic model showed a best-fit value of 0.98/2 (best-fit value/number of PhFs; Figure 1) and thus confirms that 4MCHA would have been identified by screening if it was present in the virtual chemical library. The selected PhFs (with a bias towards the *Pf*SpdS structure co-crystallized with dcAdoMet and 4MCHA, [PDB:2PT9]) derived from the MIF analyses were used to construct a shortlist of eight DPMs consisting of either three or four PhFs each. These eight DPMs were screened against the drug-like subset of the ZINC database and between 100 and 22,000 hits were identified. Eleven compounds were selected following visual inspection and filtering based on best-fit values and molecular mass. Docking was used to further filter and rank the compounds based on their binding poses and docking energies within the active site.

Similar to 4MCHA, 1-(3-ammoniopropyl)-4-methylpiperidin-1-ium (compound 1) satisfied both the HYD and positive ionizable (PI) PhFs with an additional aminopropyl chain that fits to a novel PI PhF identified within the non-attacking region of the putrescine-binding cavity (Figure 1). Compound 1 showed a reasonable best-fit value of 0.53/3 with a docking energy of −7.4 kcal/mol and reduced *Pf*SpdS activity by 17.0 ± 9.4% at 100 μM (Table 1). A binding pose where the aminopropyl chain of compound 1 traverses the catalytic centre and binds in the dcAdoMet cavity was predicted to be less favourable but could not be excluded.

Many of the hits obtained from the virtual screen against *Pf*SpdS contained either a piperidine or piperazine ring and it was therefore of interest to determine whether these rings would be able to fit into the same chemical space as the cyclohexane ring of 4MCHA. A similarity search identified a 4MCHA analogue, *trans*-4-amino-1-methylpiperidine (compound 2; Figure 1), which differs from 4MCHA by a ring nitrogen. Docking of compound 2 with its ring nitrogen in the protonated state showed a similar orientation to 4MCHA with regards to its methyl and amino group (docking score −8.2 kcal/mol; pK_a_ of *N*-methylpiperidine 10.1) [52]. However, compound 2 reduced *Pf*SpdS specific activity by only 6.9 ± 5.8% at 100 μM (Table 1) suggesting that piperidine rings are not well accommodated within the putrescine-binding cavity.

### DPM2 binding cavity

Fourteen DPMs were generated from the DPM2 binding cavity for virtual screening. The PhFs for this region were selected from both the sub-ensemble of structures and crystallographic data. PhFs selected to represent the non-attacking putrescine-binding region were biased to the data derived from *Pf*SpdS co-crystallized with 4MCHA and dcAdoMet. The virtual screens of the 14 DPMs resulted in between one and 1,800 hits for the respective DPMs, from which 24 compounds were selected for docking. Two compounds were chosen for *in vitro* testing (Additional file 1). (5*S*)-6-amino-5-[[(2*S*)-2-azaniumyl-3-(4-hydroxyphenyl) propanoyl] amino]-6-oxohexyl] azanium (compound 3) was selected based on its good best-fit value (0.91/4) and favourable docking energy (−12.5 kcal/mol). 3-[4-(3-azaniumylpropyl)piperazine-1,4-diium-1-yl]propylazanium (compound 4) showed a low best-fit value (0.15/4) but a docking score of −9.8 kcal/mol and a good binding pose as revealed by visual inspection. Compound 4 contains a piperazine ring and was predicted to bind within the non-attacking nitrogen binding site of putrescine. The other aminopropyl chain of compound 4 was predicted to bind in the same binding pocket as the aminopropyl chain of dcAdoMet. However, *in vitro* data showed that compounds 3 and 4 did not inhibit the enzyme at a 100 μM concentration (Additional file 1).

### DPM3 and DPM4 binding cavities

Ten different DPMs were constructed for the DPM3 cavity each consisting of four to six PhFs and between 0 to 1,813 hits were identified by virtual screening. Filtering and visual inspection identified seven compounds for docking. *N*’-[(2-hydroxy-4-oxocyclohexa-2,5-dien-1-ylidene)methyl]-3-(4-methylpiperazine-1,4-diium-1-yl)-propanehydrazide (compound 5) and [(2*R*)-3-azaniumyl-2-hydroxypropyl]-[(2*S*)-2-hydroxy-3-piperidin-1-ium-1-ylpropyl]-methylazanium (compound 6) represent new scaffolds for binding to the dcAdoMet cavity and were selected for *in vitro* testing. However, neither compound showed reduction in *Pf*SpdS activity (Additional file 1).

The DPM4 cavity covers the entire active site and eight DPMs consisting of four or five PhFs each, were prepared for screening. These DPMs defined AdoDATO with a best-fit value of 0.66/5 and served as a control to validate that known inhibitors of this binding cavity would have been identified. Screening resulted in 0 to 80 hits for the eight DPMs from which six compounds were selected for docking. Only [(2*S*)-6-azaniumyl-1-[[(2*S*)-6-azaniumyl-1-(naphthalen-2-ylamino)-1-oxohexan-2-yl]amino]-1-oxohexan-2-yl]azanium (compound 7) occupied the entire cavity but showed a low best-fit value of 0.14/5 with a docking energy of −10.5 kcal/mol and showed no inhibition (Additional file 1).

### Knowledge-based design of inhibitors

It was postulated that the lower IC_50_ of adenosylspermidine compared to AdoDATO [21,38] against mammalian SpdS is due to additional hydrogen bonding in the catalytic centre via the secondary amine of adenosylspermidine, which mimics the attacking nitrogen of putrescine. Binding free energy (ΔG) calculations of adenosylspermidine and an analogue, in which the nitrogen atom is replaced by a carbon atom, showed an increase of 15 kcal/mol for the latter (Figure 1; arrow). Conversely, replacing the corresponding carbon atom in AdoDATO by nitrogen reduced the binding free energy of the analogue by 35 kcal/mol (MM-GB/SA; Schrödinger; Figure 1; arrow).

**Figure 4:**
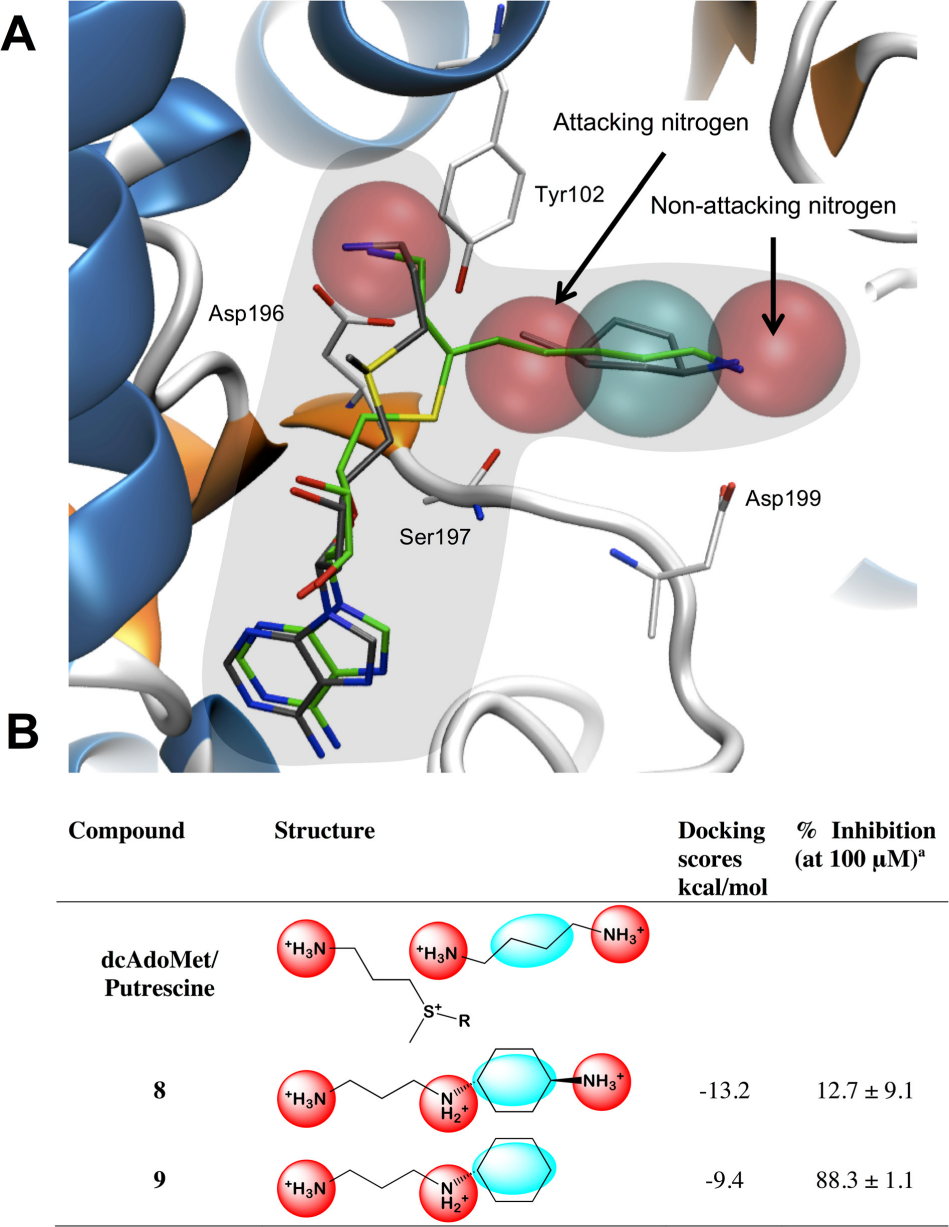
**The most important binding characteristics of the DPM2 cavity and the compounds selected to explore this region. (A)** The PhFs best describing the binding characteristics of the DPM2 cavity. The red and blue spheres represent the PI and hydrophobic features, respectively. The PhFs of AdoDATO are shown in green while those of 4MCHA and dcAdoMet are in grey. The amino acid residues in white represent those that best define the interactions described by the PhFs. From left to right the red spheres represent the PI features of the aminopropyl chain of dcAdoMet, the middle feature represents the attacking nitrogen of putrescine and on the right is the feature of the non-attacking nitrogen of putrescine. In blue is the hydrophobic feature representative of the butyl group of putrescine. **(B)** The structures of the compounds including their docking scores, selected for inhibition assays of *Pf*SpdS activity at 100 *μ*M. ^a^Results represent the percentage PfSpdS activity compared to untreated enzyme of the three independent experiments performed in duplicate. Data are given as ± SEMS, n = 3 or n = 4.

Using the above information, the DPM2 cavity was selected as a basis for the knowledge-based design of inhibitors since it includes the catalytic centre, the putrescine binding cavity and part of the dcAdoMet binding pocket (Figure 3). Preference was therefore given to small molecules able to favourably cross the electron-rich catalytic centre between the binding cavities (from the putrescine binding cavity to the dcAdoMet cavity), which contain an amine group and would promote closure of the gate-keeping loop in the presence of MTA. Knowledge-based design required compounds to match the chemical features of important PhFs that describe the DPM2 binding cavity and included the prominent PI PhF that represents the aminopropyl chain of dcAdoMet and the non-attacking nitrogen of putrescine (Figure 4A). In addition, the PI PhF that describes the attacking nitrogen of putrescine and the HYD PhF of putrescine’s butyl chain, were also included (Figure 4A).

*N*-(3-aminopropyl)-*trans*-cyclohexane-1,4-diamine (compound 8) was derived from the above considerations and represents a basic scaffold for the DPM2 cavity (Figure 4B). Docking of compound 8 showed the expected binding poses with a binding energy of −13.2 kcal/mol (Figure 4B). The cyclohexylamine moiety is predicted to bind similarly to 4MCHA with the corresponding amino group forming a hydrogen bond with Asp199 (gate-keeping loop residue). The bridging nitrogen connecting the aminopropyl chain of compound 8 to the cyclohexane ring is predicted to form two hydrogen bonds; one with Tyr102 (residue from Rossman-like fold) and another with either the backbone carbonyl group of Ser197 (gate-keeping loop residue) or interchangeably with Gln93. These hydrogen bonds are expected to reduce the binding penalty of an aliphatic carbon chain that bridges the catalytic centre as suggested by the calculations of the ΔG’s for AdoDATO and adenosylspermidine and their analogues (see above). The *in silico* model also predicted that compound 8 would cooperatively bind with MTA to facilitate closure of the gate-keeping loop. This compound reduced *Pf*SpdS activity by only 12.7 ± 9.1% at 100 μM (n = 3; Figure 4B) with an IC_50_ value of 619 ± 144 *μ*M (n = 3; Table 1).

In addition, *N*-(3-aminopropyl)-cyclohexylamine (compound 9) was identified as being similar to compound 8 differing by missing a 4-amino group (similarity search, SciFinder; Figure 4B). This compound was therefore expected to assume the same binding pose and hydrogen bond pattern as compound 8 (Figure 4B). Docking of compound 9 showed a good binding pose and a binding energy of −9.4 kcal/mol (Figure 4B). *In vitro* testing of compound 9 at 100 μM showed an 88.3 ± 1.2% (n = 5) reduction in *Pf*SpdS activity (Figure 4B) with an IC_50_ value of 7.4 ± 2.4 μM (n = 3; Table 1). This value is similar to the IC_50_ values of AdoDATO [20] and 5-amino-1-pentene (APE) [18] but higher than 4MHCA (Table 1).

### X-ray crystallography

**Figure 5:**
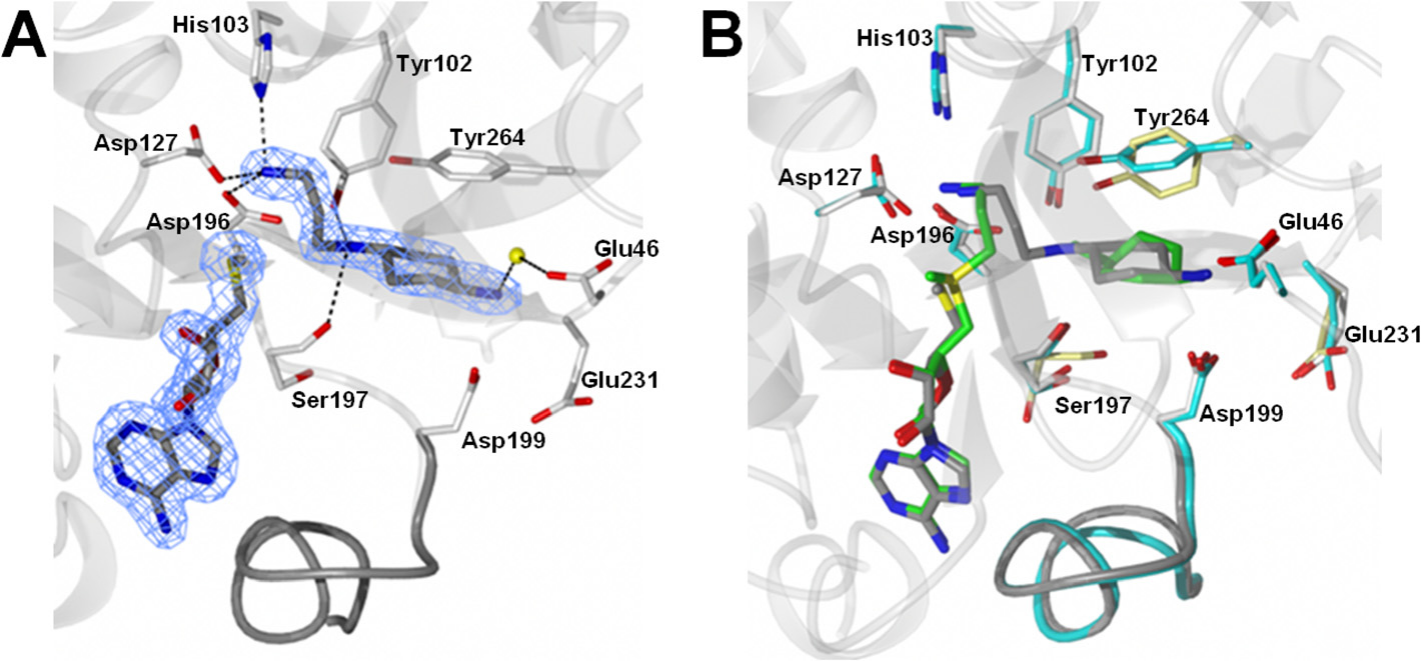
***Pf*** **SpdS co-crystallized with MTA and compound 8. (A)** The electron densities of MTA and compound 8 are shown in blue (σ = 1). The residues involved in hydrogen bonding with compound 8 are indicated with dotted lines. The gate-keeping loop covering the active site is shown as a grey ribbon with two of its residues, Ser197 and Asp199, which together with residues Glu46, Tyr102, His103, Asp127 Asp196, Glu231 and Tyr264, forms the active site. The solvent molecule involved in an interaction with compound 8 via Glu46 is shown as a yellow sphere. **(B)** MTA and compound 8 of the 3RIE structure in grey are superimposed with 4MCHA and dcAdoMet in green from the 2PT9 crystal structure. The active site residues involved in compound 8 and 4MCHA binding are shown in grey and cyan, respectively.

**Figure 6:**
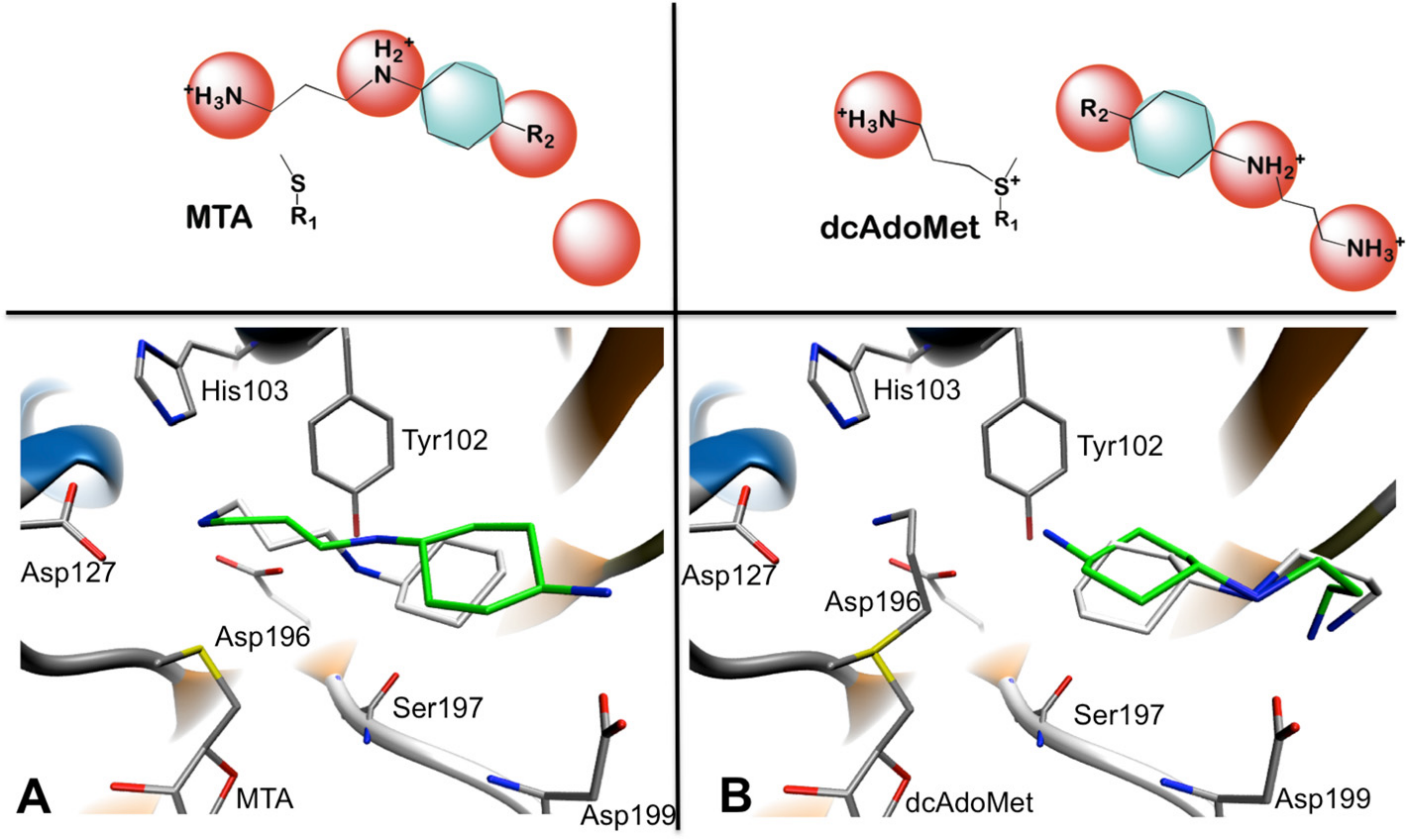
**Different binding poses that compounds 8 (green) and 9 (white) can assume upon binding with MTA and dcAdoMet, respectively. (A)** Compounds 8 and 9 traverse the catalytic centre when cooperatively bound with MTA. The aminopropyl chains of compounds 8 and 9 bind in the chemical space that are normally occupied by the aminopropyl chain of dcAdoMet. **(B)** Compound 8 and 9 assume a reverse binding pose in the presence of dcAdoMet. Compounds 8 and 9 form hydrogen bonds with Asp199 and a water molecule anchored by Ile92 and Glu46 (not shown) in the non-attacking end of the putrescine-binding cavity. An additional hydrogen bond is formed by compound 9 with Tyr102.

To experimentally determine the binding mode of compounds 8 and 9 crystallization screens were performed with a 39 amino acid N-terminal truncated *Pf*SpdS protein as described by Dufe *et al.* [20]. The purified protein was mixed with either compound 8 or 9 in the presence or absence of MTA to produce *Pf*SpdS-inhibitor-MTA or *Pf*SpdS-inhibitor complexes, respectively. Testing of numerous conditions similar to the ones previously described by Dufe *et al.* [20] for crystallization of *Pf*SpdS, yielded crystals within several days that diffracted to 1.9 Å (Figure 5). Data collection and refinement statistics for the compound 8 and MTA *Pf*SpdS crystal structure are listed in Table 3. As with all the previous structures of *Pf*SpdS, the protein crystallized in space group *C*121 with three molecules in the asymmetric unit (Table 3). Subunits B and C form a homodimer with a buried interface of 1424 Å^2^ as analysed with PISA [53]. The *Pf*SpdS structure of MTA with compound 8 [PDB:3RIE] superimposes with RMSD values of 0.31 and 0.33 Å^2^ with the ligand free [PDB:2PSS] and dcAdoMet-4MCHA [PDB:2PT9] structures, respectively (Figure 4B). *Pf*SpdS with compound 8 and MTA showed high occupancies for both ligands in all the protein molecules in the asymmetric unit. MTA is bound in the dcAdoMet binding site of *Pf*SpdS structures as previously shown ([PDB:2HTE]; [PDB:3BP7]). The binding pose of compound 8 confirmed the *in silico* predicted binding orientation of the compound within the DPM2 binding site in the presence of MTA as shown in Figures 4A and 6A. The non-attacking amine of the cyclohexane ring is hydrogen-bonded to Glu46 via a water molecule and to the side chain of Asp199. The bridging amino group is within hydrogen bonding distance of Tyr102 and Ser197 and facilitates the traversal of compound 8 across the catalytic centre. The nitrogen (epsilon) of His103 and one carboxyl oxygen each of Asp127 and Asp196 do form hydrogen bonds to the terminal aminopropyl nitrogen of compound 8 (Figure 6A). These interactions correspond to the interactions of the aminopropyl nitrogen of dcAdoMet in complex with 4MCHA. Moreover, clear electron density for the gate-keeping loop was observed when both compound 8 and MTA were present. The average B factor values for the loop residues are in the range of the overall B factors (19.30/25.46 overall/loop) and comparable with that of the structure containing AdoDATO ([PDB:2I7C]; Additional file 4). In the presence of both dcAdoMet and 4MCHA ([PDB:2PT9]; Additional file 4) the gate-keeping loop of the resolved structure also has high B values [20]. Previous studies have shown that the gate-keeping loop is disordered in the ligand free structure [PDB:2PSS] or when only MTA is present [PDB:2HTE]. Crystallization trials with compound 9 and MTA resulted in crystals with poor diffraction quality and weak density and could not be used for structure solving.

**Table 3:** **Crystallography data collection and refinements statistics**

**Data collection**	
Beamline	MAX II I911-2
Wavelength (Å)	1.04
Space group	*C*121
Unit cell (a; b; c; β) (Å)	196.8; 134.6; 48.5; 94.55°
Molecules per ASU	3
Resolution (Å)	20.0-1.9 (1.95-1.9)
Observed reflections	413845 (30068)
Unique reflections	98147 (7216)
Completeness (%)	99.8 (100)
Multiplicity	4.2 (4.2)
*</I >* \ < σ>	18.47 (3.92)
*R*_meas_ (%)	5.9 (41.1)
**Refinement statistics**	
Resolution	20-1.9 (1.94-1.89)
Reflections	93239 (5053)
*R*_cryst_ (%)	17.8 (29.6)
*R*_free_ (%)	21.0 (30/7)
Atoms	7493
Non-hydrogen	6876
Water	617
Average B-factor (Å^2^)	25.46
RMSD from ideal	
Bond lengths (Å)	0.029
Bond angles (°)	2.1
Ramachandran statistics (%)^*a*^	
Favoured	97.01
Outliers	0.12

Values in parenthesis are for the high resolution shell.

^*a*^Ramachandran statistics were calculated using Molprobity [47].

### Proposed alternative binding poses for compounds 8 and 9

**Figure 7:**
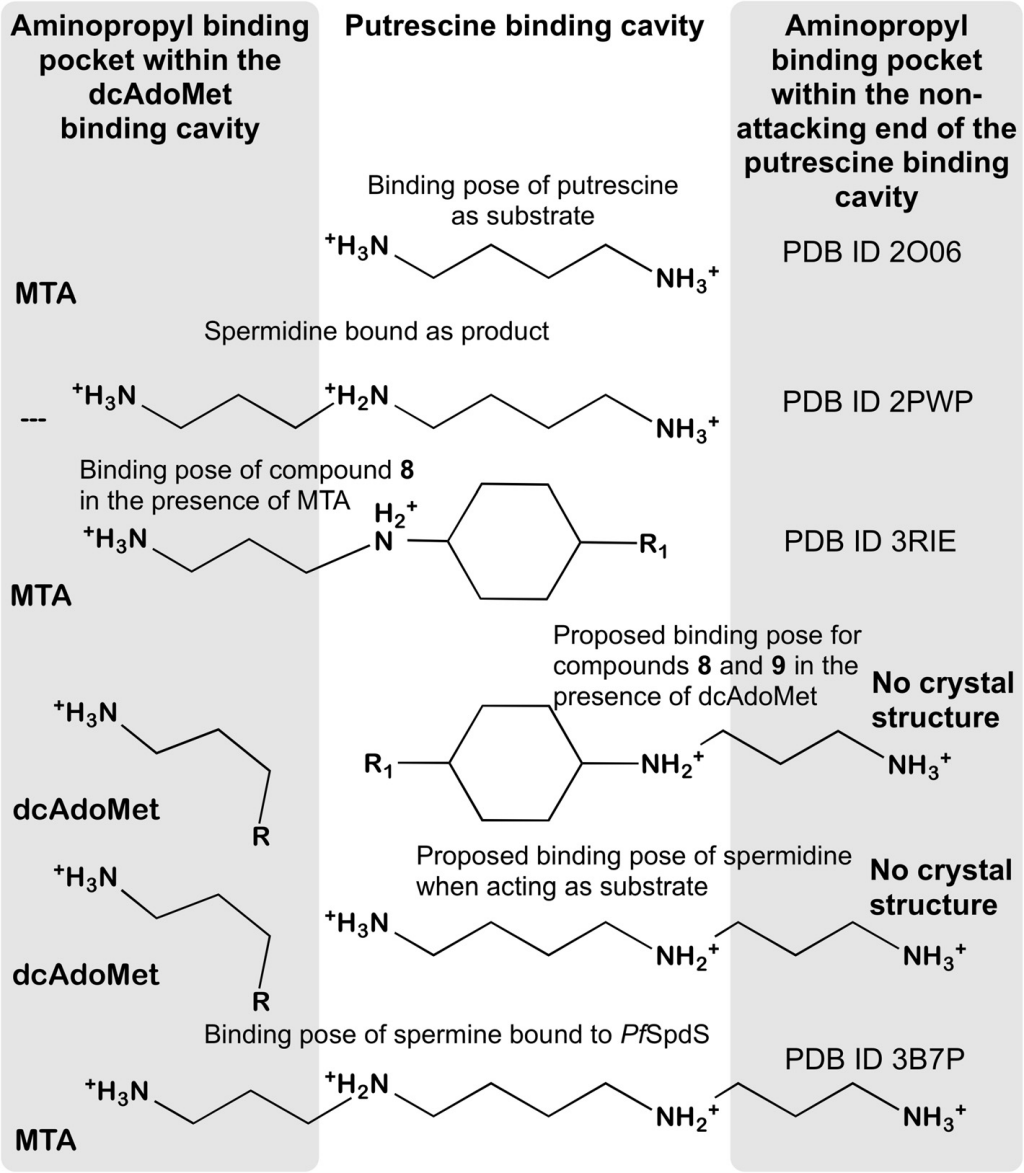
**A schematic representation of the binding poses of putrescine spermidine, spermine, compound 8 and compound 9 to SpdS.** The grey highlighted columns depict the two different binding pockets for the aminopropyl chains of spermidine, spermine, compound 8 and compound 9. The grey column on the left depicts the aminopropyl binding pocket associated with the dcAdoMet binding cavity. The grey column on the right depicts the additional aminopropyl binding pocket located in the non-attacking end of the putrescine binding cavity responsible for the ability of *Pf*SpdS to produce spermine. The white column depicts the putrescine binding cavity and the corresponding moieties of the analogues depicted in this Figure. PDB IDs are given when the binding poses of compounds were from crystal structures. When ligands are resolved in the presence of MTA or dcAdoMet they are indicated in the aminopropyl binding pocket column of dcAdoMet (grey column on the left). For compounds resolved with dcAdoMet their aminopropyl chains are displayed in their binding cavities. R_1_ = NH_3_^+^ is compound 8; R_1_ = H is compound 9.

The crystal structure with *Pf*SpdS in complex with MTA and compound 8 confirmed the *in silico* predicted binding poses of these two ligands in the active site. Since no resolved crystal structure of *Pf*SpdS with bound MTA and compound 9 could be obtained, there is no structural explanation for the unexpected discrepancy in the IC_50_ values between compounds 8 and 9. However, Shirahata *et al*. identified compound 9 as an inhibitor of human spermine synthase but not of human SpdS [22]. *Pf*SpdS also accepts spermidine as secondary substrate to synthesize spermine and Shirahata’s results therefore suggest that compounds 8 and 9 bind in the active site in a reversed orientation, i.e., aminopropyl chains facing the non-attacking side of putrescine binding cavity, with dcAdoMet present instead of MTA (Figure 6). The implication of this mode of binding by compounds 8 and 9 is that these ligands must compete with both putrescine and spermidine for binding in presence of dcAdoMet, which is supported by preliminary kinetic experiments showing a mixed-type competitive inhibition between compound 9 and putrescine (K_*i*_ of 2.8 μM; Additional file 5). A schematic representation of the binding poses of compounds 8 and 9 in *Pf*SpdS (in the presence of dcAdoMet) relative to those of spermidine as substrate and product as well as spermine is presented in Figure 7. Docking of compound 9 in the reverse orientation with dcAdoMet instead of MTA furthermore showed it to be the stronger binder (−8.5 kcal/mol) of *Pf*SpdS (Figure 6) than compound 8 (−7.1 kcal/mol). Most notable is the increase in the predicted binding penalty energy due to strain between the receptor in the complex and the receptor in the free state for compound 8 in comparison to compound 9 (11.3 kcal/mol and 3.8 kcal/mol respectively, using MM-GB/SA). These results would explain the significant differences in the IC_50_ values of compounds 8 and 9. In addition, compound 8 bound in the reverse orientation, i.e., with the primary amine of the cyclohexyl ring facing the catalytic centre, also acts as a pseudo-substrate for *Pf*SpdS (JS *et al.,* unpublished results). This in effect would lower its concentration as catalysis proceeds.

Attempts to grow diffraction quality *Pf*SpdS crystals with bound compound 9 and dcAdoMet were unsuccessful. Against this background further insight into the putrescine/spermidine binding pocket was obtained from the *Pf*SpdS structure co-crystallized with spermidine [PDB:2PWP] and spermine [PDB:3B7P]. Compound 8 assumes a similar binding orientation [PDB:3RIE] to that of spermidine in the presence of MTA [PDB:4CXM] with its aminopropyl chain also located in the dcAdoMet cavity and similar interactions with the catalytic centre. Interestingly, one of the aminopropyl chains of spermine co-crystallized with MTA [PDB:3B7P] is located in the dcAdoMet binding cavity and its putrescine moiety located in the putrescine binding cavity similar to spermidine co-crystallized with *Pf*SpdS [PDB:2PWP]. The other or second aminopropyl chain of spermine is located in the non-attacking end of the putrescine-binding cavity and show two distinct binding poses in the three different protein chains. The aminopropyl chains of compounds 8 and 9 when bound in the reverse orientation are similar to the dominating binding pose of spermine noticed in two of the three subunits [PDB:3B7P] (Additional files 6 and 7). Noteworthy, two residues Glu231 and His236, showed altered conformations that facilitate the accommodation of the aminopropyl chain of spermine in the non-attacking end of the putrescine-binding cavity.

Finally, a comparison between the proposed binding poses of compounds 8 and 9 in *Pf*SpdS to that of spermine crystallized with *Hs*SpmS demonstrated distinct binding cavities for the aminopropyl chains in the non-attacking end of the putrescine/spermidine binding cavity (Additional files 6 and 7). Docking of compounds 8 and 9 (−8.8 and −8.7 kcal/mol, respectively) to *Hs*SpmS [PDB:3C6K] showed that their aminopropyl chains bind in the same orientation and cavity to that of the enzyme co-crystallized with spermidine. These observations suggest that compounds may be designed with selectivity for *Pf*SpdS and not *Hs*SpmS by exploring the extra aminopropyl cavity that allows *Pf*SpdS to accommodate spermidine for synthesis of spermine.

## Discussion

The most effective inhibitors for SpdS were derived following a ligand-based approach and include 4MCHA, AdoDATO and adenosylspermidine. 4MCHA is the most potent inhibitor of *Pf*SpdS with an IC_50_ value of 1.4 μM followed by AdoDATO with an IC_50_ value of 8.5 μM (Table 1) [18,20]. The 170-fold lower IC_50_ value of AdoDATO for mammalian SpdS compared to the *P. falciparum* enzyme has been suggested to be due to a higher K_m_ value of the latter enzyme for dcAdoMet (35 μM) compared to the mammalian enzyme (7–25 μM) [18,20,38]. The active site of *Pf*SpdS accommodates dcAdoMet and either putrescine or spermidine in the second substrate-binding cavity. The latter substrate is converted into spermine and accounts for 15% of the enzyme’s activity [18]. In addition, binding of ligands into the putrescine binding cavity is suggested to occur subsequent to binding of ligands into the dcAdoMet binding cavity [20]. In the first structure-based drug design study for *Pf*SpdS, AdoDATO was used to select PhFs for the generation of pharmacophore models [23]. Screening of the models against an in-house chemical library identified several binders to the active site but did not yield any promising inhibitory compounds. Re-evaluation of the active site suggested that its size and shape are unfavourable for a standard virtual screen and was, therefore, divided in this study into four binding regions as shown in Figure 3. ‘Dynamic’ receptor-based pharmacophore models (DPM1-4; Figure 3) were developed for each region and information obtained from previous studies as well as new information derived in this study was incorporated [22,54]. The methodology used here uniquely also included protein flexibility, which was captured during phase space sampling and represented as a sub-ensemble of structures. Importantly, this approach highlighted a conformational change in Gln229 between the ligand free- and ligand-bound states of *Pf*SpdS, which significantly alters the binding characteristics of the putrescine-binding cavity (Additional file 3). Potential inhibitors were identified from the derived pharmacophore models by a combination of virtual screening of the ZINC database, knowledge-based design and similarity searches. Compounds that matched features of interest were subsequently docked into the *Pf*SpdS active site before being selected for inhibition assays.

By exploring the DPM1 cavity compound 1 (Figure 1) was identified and predicted to bind with its aminopropyl chain protruding into the non-attacking side of the putrescine-binding cavity and the piperidine ring overlapping with the cyclohexane ring of 4MCHA. The possibility that it binds in a reversed orientation as an explanation for the weak inhibition of *Pf*SpdS at 100 μM, was excluded due to unfavourable docking scores. This result suggested that a piperidine ring is not easily accommodated within the DPM1 cavity when the ring nitrogen faces the catalytic centre and is supported by results obtained for compound 2.

No noteworthy inhibitory compounds were identified by virtual screening of the binding cavities of DPM2-4. The result for the DPM4 binding cavity was however not surprising considering the dimensions and shape of the binding cavities described by the pharmacophore models (Figure 3) and the limited chemical space covered by the drug-like subset of the ZINC database.

The gate-keeping loop (residues 197–205) that covers the active site has been proposed to play an important role in putrescine-binding as well as inhibition of SpdS by 4MCHA [18,50]. Putrescine forms hydrogen bonds with loop residues Ser197 and Asp199 at the attacking and non-attacking ends of the putrescine-binding cavity of *Pf*SpdS, respectively. 4MCHA binds in the putrescine cavity by hydrogen bonding to loop residue Asp199 but does not form hydrogen bonds at the catalytic centre. It was therefore postulated that compounds that traverse the catalytic centre and bind to the gate-keeping loop at more than one site would result in an extended closed state of the loop and an improved inhibitor of *Pf*SpdS compared to 4MCHA. The SpdS inhibitor AdoDATO is a ~3-7-fold less efficient inhibitor of mammalian SpdS than adenosylspermidine (Table 1) [21,38]. The aliphatic aminopentyl chain of both multi-substrate analogues traverses the electron rich catalytic centre and interacts with loop residue Asp199 at the non-attacking end of putrescine, similar to 4MCHA. Binding free energy calculations here show that the lower IC_50_ value of adenosylspermidine compared to AdoDATO is most likely due to the secondary amine in the aliphatic aminopentyl chain of adenosylspermidine (Figure 1; arrow), which permits additional hydrogen bonding with Ser197 (gate-keeping loop) and Tyr102. Binding free energy calculations performed on *Pf*SpdS showed similar results.

Prompted by the above observations, small molecule inhibitors that directly interact with the gate-keeping loop were knowledge-based designed, based on four PhFs identified in the DPM2 binding cavity (Figure 4A). Compounds 8 and 9 were predicted to assume binding poses in which they cross the catalytic centre with their aminopropyl chains bound within the dcAdoMet-binding cavity provided that MTA is present (Figure 6A). This was confirmed for compound 8 by a crystal structure co-crystallized with MTA (Figure 5) with similar B factor values for the gate-keeping loop to those of the *Pf*SpdS crystal structure resolved with AdoDATO (Additional file 4).

It was envisioned that once dcAdoMet is converted into MTA, binding of compounds 8 and 9 would be promoted, especially of compound 8 due to additional hydrogen bond formation with the gate-keeping loop, which would prolong its closure. However, the 100-fold weaker IC_50_ of compound 8 compared to compound 9 does not support this novel hypothesis. Pre-incubation of *Pf*SpdS with MTA up to concentrations of 100 μM prior to assays furthermore did not change the inhibitory capacity of these compounds (AIL *et al.* unpublished results). Preliminary kinetic experiments with *Pf*SpdS however indicated a mixed-type competitive inhibition between compound 9 and putrescine with a K_*i*_ of 2.8 μM (Additional file 5). Shirahata *et al*. [22] identified compound 9 as an inhibitor of mammalian spermine synthase, which however did not inhibit mammalian spermidine synthase. A plausible explanation for the results presented here, therefore, is that competition between the aminopropyl chains of compounds 8 and 9 with that of dcAdoMet (Figures 5 and 6) may force their binding in a reversed orientation with their chains now facing the non-attacking end of the putrescine binding cavity, i.e., acting as spermidine analogues. Docking results with dcAdoMet instead of MTA indicated a higher binding affinity for compound 9 compared to compound 8 when their binding orientation was reversed (Figures 6 and 7). Moreover, a three-fold increase in receptor (*Pf*SpdS) strain was predicted for compound 8 in comparison to compound 9. Taken together, these results would explain the observed disparity between the IC_50_ values of compounds 8 and 9. An earlier report has shown that the diamine substrate, trans-1,4-diaminocyclohexane, acts as a pseudo-substrate (~12%) for mammalian SpdS [22]. Similar results were found for compound 8 (JS *et al.,* unpublished results), which in consequence would lower its effective inhibitory capacity as catalysis proceeds since it accepts an aminopropyl chain from dcAdoMet, which diminishes its residence time within the binding cavity. Importantly, no evidence was found for compound 9 acting as a pseudo-substrate. It is accepted that some percentage of compounds 8 and 9 may also bind with their chains facing the catalytic centre, albeit briefly as catalysis proceeds and MTA is produced.

Interestingly, a comparison of the *Hs*SpmS [PDB:3C6M] and *Pf*SpdS [PDB:3B7P] structures resolved with spermine show two distinct binding pockets for the aminopropyl chain of spermine at the non-attacking end of the putrescine/spermidine binding cavity. The current docking studies showed that the aminopropyl chain of compound 9 bind to these distinct pockets, when docked to *Pf*SpdS or *Hs*SpmS, which could be explored for inhibitor selectivity between these enzymes.

## Conclusions

This study brings together an array of chemical, biological, biophysical and molecular modeling techniques capturing and explaining the most important characteristics of ligand binding to *Pf*SpdS. Although the computational studies correctly predicted the lowest binding energy pose of *Pf*SpdS inhibitors no clear correlations were observed between the docking scores and inhibitory values of potential and known inhibitors of *Pf*SpdS. The discrepancies could be explained by challenges when docking positively charged flexible polyamines and when working with multi-substrate enzymes. It was essential to incorporate previously reported medicinal chemistry results in this study to guide decision making in the lead discovery process [21,22,54-56]. As a result an *in silico* predicted binding pose of compound 8 in the presence of the end product, MTA, was confirmed by crystallography studies. Noteworthy is that compound 8 had an unexpected 100-fold lower IC_50_ value than compound 9 despite its similarity, which resulted in the re-evaluation of the initial inhibition hypothesis. Previously, Shirahata *et al.* showed compound 9 to be a potent inhibitor of mammalian spermine synthase but not of mammalian SpdS [22,54]. In this study however, compound 9 was identified as a *Pf*SpdS inhibitor with an IC_50_ comparable to the most potent known inhibitors of *Pf*SpdS. Because compound 9 did not inhibit mammalian SpdS it makes it the only known inhibitor specific for *Pf*SpdS. Its specificity is attributed to the ability of *Pf*SpdS to alternatively accommodate spermidine to putrescine as substrate for spermine synthesis. The potency of compound 9 was a surprising result given that the rate of spermine synthesis by *Pf*SpdS is only 15% of the rate of spermidine synthesis [18]. These results provide new insights and opportunities, especially the distinctive binding of the aminopropyl chain of compound 9 to the non-attacking end of the putrescine/spermidine binding cavity, to be explored for the design of selective inhibitors of *Pf*SpdS.

### Accession code

Coordinate and structure factor files for the *Pf*SpdS complex [PDB: 3RIE] were deposited at the Protein Data Bank.

## Additional files

Additional file 1:
**Additional compounds identified from virtual screening that were docked and tested** ***in vitro*** **against** ***Pf*** **SpdS.**
Additional file 2:
**Phase space sampling: A 5 ns molecular dynamics (MD) simulation used to capture the flexibility of the active site.**
Additional file 3:
**An illustration of the conformational change Gln229 undergoes upon binding of 4MCHA and AdoDATO as well as putrescine.**
Additional file 4:
**Average B factor values for the C**_**α**_ **backbone and gate-keeping loop of three different** ***Pf*** **SpdS crystal structures.**
Additional file 5:
**Inhibition kinetics of** ***Pf*** **SpdS treated with compound 9.**
Additional file 6:
**Superimposition of the human spermine synthase (grey ribbon) and** ***Pf*** **SpdS (blue ribbon).**
Additional file 7:
**Two different binding poses spermine assumes when co-crystallized with MTA within** ***Pf*** **SpdS.**

## Abbreviations

AdoDATO: *S*-adenosyl-1,8-diamino-3-thio-octane
AdoMetDC: *S*-adenosylmethionine decarboxylase
APA: 3-aminooxy-1-aminopropane
APE: 5-amino-1-pentene
dcAdoMet: decarboxylated *S*-adenosylmethionine
DPM: Dynamic pharmacophore model
EV: Exclusion volume
HBA: Hydrogen bond acceptor
HBD: Hydrogen bond donor
HYD: Hydrophobic
4MCHA: *trans*-4-methylcyclohexylamine
MIF: Molecular interaction field
MM-GB/SA: Molecular mechanics generalized born/surface accessible
MTA: 5′-methylthioadenosine
ns: nanosecond
ODC: Ornithine decarboxylase
PBC: Periodic boundary conditions
PhF: Pharmacophore feature
PI: Positive ionizable
PME: Particle mesh Ewald summation
ps: picosecond
SD: Steepest decent
SpdS: Spermidine synthase
SpmS: Spermine synthase
