# Supplementary material for: A novel inhibitor of Plasmodium falciparum spermidine synthase: a twist in the tail
Source: Malar J. 2015 Feb 5;14:54. doi: 10.1186/s12936-015-0572-z (PMC4342090; doi:10.1186/s12936-015-0572-z)
Supplement: Additional file 1: — Additional compounds identified from virtual screening that were docked and tested in vitro against Pf SpdS. [file 12936_2015_572_MOESM1_ESM.pdf]

Additional file 1 Additional compounds identified from virtual screening that were docked and tested *in vitro* against *Pf*SpdS.

Compound	Structure	Best Fit Value/number of PhFs	Docking scores (kcal/mol)	% Inhibition (at 100 μM)[a]
DPM2				
3	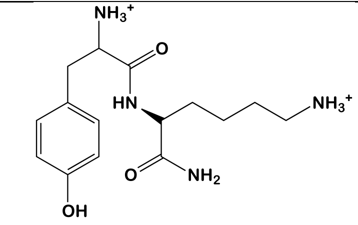	0.91/4	-12.5	No Inhibition
4	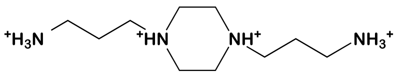	0.15/4	-9.8	No Inhibition
DPM3				
5	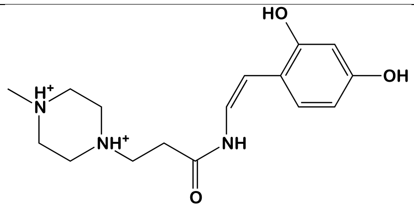	0.10/5	-9.0	No Inhibition
6	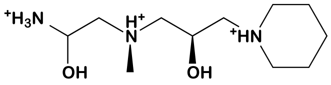	0.18/4	-9.7	No Inhibition
DPM4				
7	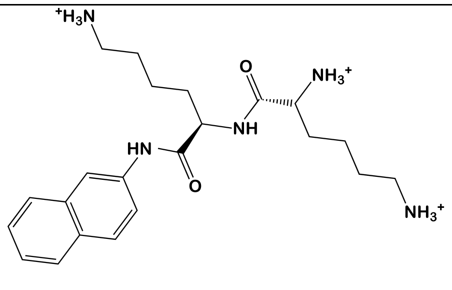	0.14/5	-10.5	No Inhibition

[a] Results represent inhibition of PfSpdS activity compared to untreated enzyme of three independent experiments performed in duplicate.
