# Supplementary material for: A novel inhibitor of Plasmodium falciparum spermidine synthase: a twist in the tail
Source: Malar J. 2015 Feb 5;14:54. doi: 10.1186/s12936-015-0572-z (PMC4342090; doi:10.1186/s12936-015-0572-z)
Supplement: Additional file 2: — Phase space sampling: A 5 ns molecular dynamics (MD) simulation used to capture the flexibility of the active site. [file 12936_2015_572_MOESM2_ESM.pdf]

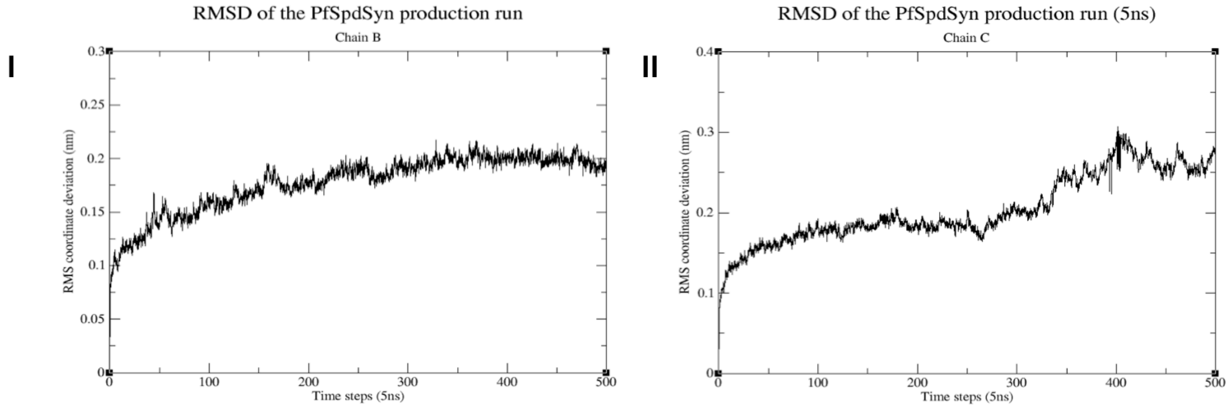

Additional file 2 Phase space sampling: A 5 ns molecular dynamics (MD) simulation used to capture the flexibility of the active site. The MD trajectory was clustered to find the best representative structures. A sub-ensemble of structures was selected from clustering and are representative of 96% of the sampled phase space. A 5 ns MD simulation of the ligand free enzyme showed similar RMSDs (root-mean-square deviation) for subunits B and C in the first 2.5 ns. A slight increase was observed in the backbone RMSD of subunit C (II) over the last 2.5 ns compared to subunit B (I) due to an increase in movement of the gate-keeping loop.
