# Supplementary material for: A novel inhibitor of Plasmodium falciparum spermidine synthase: a twist in the tail
Source: Malar J. 2015 Feb 5;14:54. doi: 10.1186/s12936-015-0572-z (PMC4342090; doi:10.1186/s12936-015-0572-z)
Supplement: Additional file 3: — An illustration of the conformational change Gln229 undergoes upon binding of 4MCHA and AdoDATO as well as putrescine. [file 12936_2015_572_MOESM3_ESM.pdf]

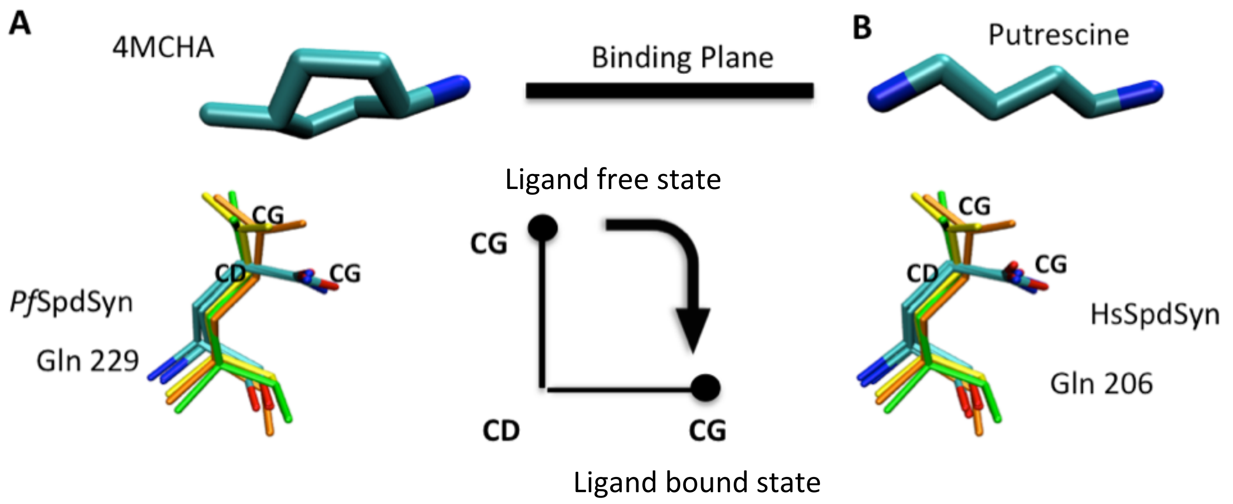

Additional file 3 An illustration of the conformational change Gln229 undergoes upon binding of 4MCHA and AdoDATO as well as putrescine. For reasons of simplicity not all the residues from the clustered structures nor the complete structures are displayed. A schematic representation of the conformational change that occurs between the ligand free and ligand bound-state for Gln229 is shown in black. It is explained using the orientation of the CD-CG bond of Gln229 with reference to an assigned binding plane (black) for 4MCHA and putrescine. **A** 4MCHA and Gln229 of the crystal structure (PDB ID 2PT9) are shown in cyan. The green, yellow and orange residues represent Clus1B, Clus2B and Clus5B, respectively, and illustrate the different conformations Gln229 adopt during the MD simulation (ligand free state). **B** Putrescine co-crystallized with the human SpdS (PDB ID 2O06) and the conformational change of Gln206, which corresponds to Gln229 of *Pf*SpdS, is shown in cyan. The Gln229 residues of *Pf*SpdS from Clus1B, Clus2B and Clus5B are represented in green, yellow and orange respectively, and are shown to highlight the differences in orientation of Gln206 within the active site of both the ligand free and bound states of SpdS.
