# Supplementary material for: A novel inhibitor of Plasmodium falciparum spermidine synthase: a twist in the tail
Source: Malar J. 2015 Feb 5;14:54. doi: 10.1186/s12936-015-0572-z (PMC4342090; doi:10.1186/s12936-015-0572-z)
Supplement: Additional file 4: — Average B factor values for the C α backbone and gate-keeping loop of three different Pf SpdS crystal structures. [file 12936_2015_572_MOESM4_ESM.pdf]

**Additional file 4** Average B factor values for the $C_\alpha$ backbone and gate-keeping loop of three different *Pf*SpdS crystal structures.

| Protein/Subunit | A | | B | | C | |
|---|---|---|---|---|---|---|
| Structures - Resolution | Overall | Loop | Overall | Loop | Overall | Loop |
| 2I7C-AdoDATO-1.71Å | 27.74 | 32.74 | 22.67 | 30.28 | 19.40 | 22.42 |
| 2PT9-4MCHA-dcAdoMet – 2.2Å | 38.76 | 49.35 | 33.36 | 46.14 | 30.90 | 38.61 |
| 3RIE-compound 8-MTA-1.9Å | 27.98 | 33.00 | 22.32 | 29.83 | 19.30 | 21.58 |
