# Supplementary material for: A novel inhibitor of Plasmodium falciparum spermidine synthase: a twist in the tail
Source: Malar J. 2015 Feb 5;14:54. doi: 10.1186/s12936-015-0572-z (PMC4342090; doi:10.1186/s12936-015-0572-z)
Supplement: Additional file 5: — Inhibition kinetics of Pf SpdS treated with compound 9. [file 12936_2015_572_MOESM5_ESM.pdf]

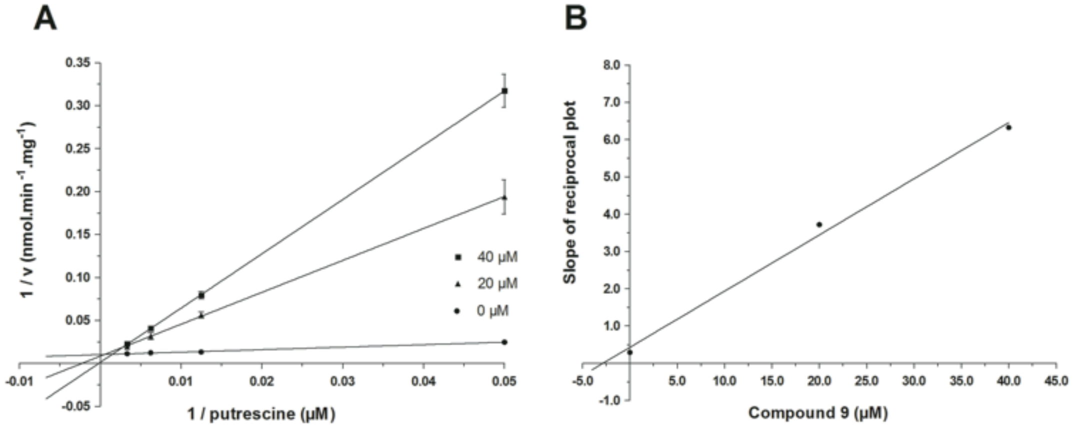

Additional file 5 Inhibition kinetics of *Pf*SpdS treated with compound **9**. (A) Lineweaver-Burk plot and (B) secondary Lineweaver-Burk plot of the slopes obtained from the plot in (A) versus inhibitor concentration to determine K_i. Results are shown as ± S.E.M obtained from five experiments performed in duplicate.
