# Supplementary material for: A novel inhibitor of Plasmodium falciparum spermidine synthase: a twist in the tail
Source: Malar J. 2015 Feb 5;14:54. doi: 10.1186/s12936-015-0572-z (PMC4342090; doi:10.1186/s12936-015-0572-z)
Supplement: Additional file 6: — Superimposition of the human spermine synthase (grey ribbon) and Pf SpdS (blue ribbon). [file 12936_2015_572_MOESM6_ESM.pdf]

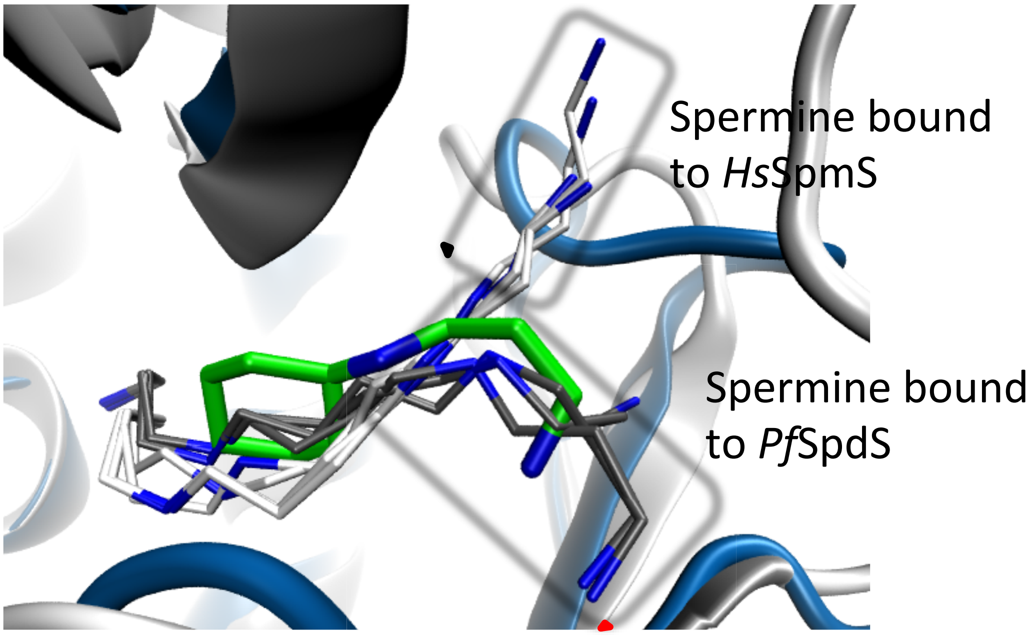

Additional file 6 Superimposition of the human spermine synthase (grey ribbon) and *Pf*SpdS (blue ribbon). The black box highlights the binding cavity occupied by spermine (white sticks) co-crystallized within the human spermine synthase [PDB:3C6M]. The red box highlights a distinct binding cavity that the aminopropyl chain of spermine (grey sticks) occupies when co-crystallized with MTA in *Pf*SpdS [PDB:3B7P]. The aminopropyl chain of compound 9 (green sticks) when docked into *Pf*SpdS [PDB:2I7C] binds in the same cavity as spermine. The slight deviation of orientation of the aminopropyl chains in *Pf*SpdS is due to the different PDB structures used in docking [PDB:2I7C].
