# Supplementary material for: A novel inhibitor of Plasmodium falciparum spermidine synthase: a twist in the tail
Source: Malar J. 2015 Feb 5;14:54. doi: 10.1186/s12936-015-0572-z (PMC4342090; doi:10.1186/s12936-015-0572-z)
Supplement: Additional file 7: — Two different binding poses spermine assumes when co-crystallized with MTA within Pf SpdS. [file 12936_2015_572_MOESM7_ESM.pdf]

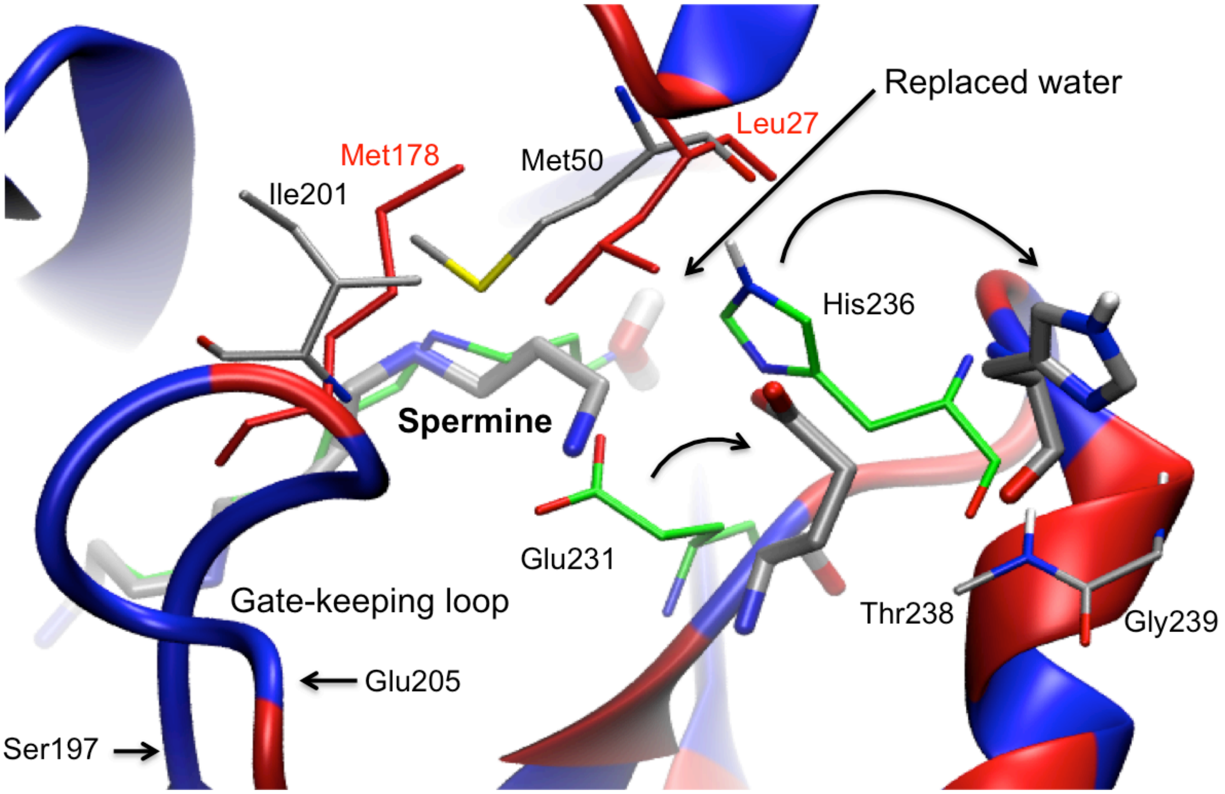

Additional file 7 Two different binding poses spermine assumes when co-crystallized with MTA within *Pf*SpdS [PDB 3B7P]. The conformation of spermine illustrated by green colored carbons displaces a water molecule found in all *Pf*SpdS crystal structures. The conformation of spermine illustrated by grey colored carbons represents the dominant orientation and is similar to that proposed for compound **9**. The conformational changes that Glu231 and His236 undergo are shown highlighted by arrows depicting the conformation they normally assume illustrated in green and the altered conformations adopted in grey.
